# *Rickettsia parkeri* infection modulates the sialome and ovariome of the Gulf coast tick, *Amblyomma maculatum*

**DOI:** 10.3389/fmicb.2022.1023980

**Published:** 2022-11-10

**Authors:** Melina Garcia Guizzo, Khemraj Budachetri, Abdulsalam Adegoke, Jose M. C. Ribeiro, Shahid Karim

**Affiliations:** ^1^Vector Biology Section, Laboratory of Malaria and Vector Research, National Institute of Allergy and Infectious Diseases, National Institutes of Health, Rockville, MD, United States; ^2^School of Biological, Environmental, and Earth Sciences, The University of Southern Mississippi, Hattiesburg, MS, United States

**Keywords:** tick, salivary glands, transcriptome, ovary, *Rickettsia parkeri*, *Amblyomma maculatum*, hematophagy, *Midichloria mitochondrii*

## Abstract

The Gulf Coast tick, *Amblyomma maculatum*, is a vector of several tick-borne pathogens, including *Rickettsia parkeri*. The ability of *R*. *parkeri* to persist within the tick population through transovarial and transstadial transmission, without apparently harming the ticks, contributes to the pathogen’s perpetuation in the tick population. Previous studies have shown that the *R*. *parkeri* load in *A*. *maculatum* is regulated by the tick tissues’ oxidant/antioxidant balance and the non-pathogenic tick microbiome. To obtain further insights into the interaction between tick and pathogen, we performed a bulk RNA-Seq for differential transcriptomic analysis of ovaries and salivary glands from *R*. *parkeri*-infected and uninfected ticks over the feeding course on a host. The most differentially expressed functional category was of bacterial origin, exhibiting a massive overexpression of bacterial transcripts in response to the *R*. *parkeri* infection. *Candidatus* Midichloria mitochondrii and bacteria from the genus Rickettsia were mainly responsible for the overexpression of bacterial transcripts. Host genes were also modulated in *R*. *parkeri*-infected tick organs. A similar number of host transcripts from all analyzed functional categories was negatively and positively modulated, revealing a global alteration of the *A*. *maculatum* transcriptome in response to pathogen infection. *R*. *parkeri* infection led to an increase in salivary transcripts involved in blood feeding success as well as a decrease in ovarian immune transcripts. We hypothesize that these transcriptional alterations facilitate pathogen persistence and transmission within tick population.

## Introduction

Ticks are obligate hematophagous parasites with a wide range of hosts capable of transmitting a variety of pathogens to humans and animals (de la Fuente et al., 2008). Ticks also harbor a diverse non-pathogenic microbiome, typically dominated by tick endosymbionts (Ponnusamy et al., 2014; Guizzo et al., 2020; Díaz-Sánchez et al., 2021; Lejal et al., 2021). Tick pathogens modulate their host’s metabolism to allow them to multiply and persist within the tick before being transmitted (Cabezas-Cruz et al., 2019; Kurokawa et al., 2020). In addition to evading tick defense mechanisms, pathogenic microorganisms must also compete with the non-pathogenic microbiome, which may impact pathogen multiplication and transmission (Narasimhan et al., 2014; Bonnet and Pollet, 2020). The Gulf Coast tick, *Amblyomma maculatum* is a vector of *Rickettsia parkeri*, which causes a febrile infection in humans (Sumner et al., 2007; Paddock et al., 2008; Cumbie et al., 2020), and also of *Hepatozoon americanum*, a pathogen of dogs (Mathew et al., 1998, 1999; Ewing et al., 2002). The distribution of *A*. *maculatum* extends from the southeastern states of the United States, bordering the Gulf of Mexico, into Mexico and several other Central and South American countries. In the past decades, it has extended Northwards and to the West in the United States, including the states of Arkansas, Oklahoma, Kansas, and southwestern Tennessee (Sonenshine, 2018).

*Rickettsia parkeri* infects *A*. *maculatum* ticks leading to an efficient transovarial and transstadial transmission without apparent harm to the ticks (Wright et al., 2015). *R*. *parkeri* is also transmitted to vertebrate hosts through tick saliva (Banajee et al., 2016). While infected, these mammalian hosts may transmit *R*. *parkeri* back to ticks, thus completing the horizontal transmission pathway of *R*. *parkeri* survival. Recent studies indicate that the rickettsial load in *A*. *maculatum* is regulated by the oxidant/antioxidant balance within tick tissues (Adamson et al., 2013; Budachetri et al., 2017a,b) and by the concurrent non-pathogenic tick microbiome (Budachetri et al., 2014, 2018).

The ability of *R*. *parkeri* to be transmitted transstadially and transovarially allows its persistence in the tick population. Therefore, the interaction between *R*. *parkeri* and tick tissues is critical for the pathogen’s continued vertical and horizontal transmission. In this study we examined the salivary and ovary transcriptomes of adult female *A*. *maculatum* ticks infected by *R*. *parkeri* to gain insights into the interactions of the pathogenic bacteria with its tick host and the endosymbiont microbiome. Our observations suggest that infection with *R*. *parkeri* leads to transcriptional changes in the host that favor its transmission and its perpetuation in the tick population.

## Materials and methods

### Ticks

*Rickettsia parkeri* infected (Rp+) and *R*. *parkeri* free (Rp-) Gulf coast tick (*A*. *maculatum*) colonies were maintained at the University of Southern Mississippi (United States) according to established methods (Patrick and Hair, 1975). Ticks were kept at room temperature under approximately 90% relative humidity and a 14 h light/10 h dark photoperiod before infestation on a sheep. Adult ticks were blood-fed on sheep and removed 2–7 days post-infestation (dpi) of their hosts, depending on the experimental protocol. The Institutional Animal Care and Use Committee at the University of Southern Mississippi approved all the protocols (#15101501.2 & 17191206.1) before the experiments commenced.

### Immunolocalization assay

The infection status of *R*. *parkeri* infected (Rp+) ticks was confirmed by immunolocalization. This was done using unfed and partially fed salivary glands and ovarian tissues. Dissected tissues were fixed in 4% PFA and 4% sucrose diluted in 1X PBS and kept at 4°C until needed. Fixed samples were washed three times in 1X PBS prior to permeabilization. Samples were permeabilized in 0.25% Triton X-100 in PBS for 30 min followed by blocking in 2% BSA in PBS for an additional 1 h. Tissues were incubated overnight at 4°C with anti-*Rickettsia* antibody (M14-13, 1:500, kindly provided by Dr. Ted Hackstadt) that recognizes *R*. *parkeri* in 1X PBS containing 2% BSA. This was followed by incubation in the dark with Alexa-Fluor 568 goat anti-mouse secondary antibody (1,500, Invitrogen, Thermo Fisher Scientific, Eugene, Oregon, United States) in 1X PBS containing 2% BSA. Samples were washed three times to remove unbound antibodies and mounted on glass slides using VECTASHIELD antifade mounting medium with DAPI (Vector Laboratories Inc., Burlingame, CA, United States).

### Image acquisition

A Leica STELLARIS STED confocal microscope was used to capture *R*. *parkeri* images in tick tissues. The 405 UV laser was used to acquire the DAPI channel while the tunable white light laser (WLL) was used to capture the Alexa-Fluor channel. For all images captured, a z-stack of the images consisting of 150–250 slices was compiled. The proprietary Leica built-in postprocessing plugin was used to deconvolute and carry out lightning processing. All images were exported as acquired and compiled in PowerPoint.

### Tissue dissections

Salivary glands from *R*. *parkeri*-infected and non-infected adult female ticks were dissected from starving ticks and from ticks that fed on sheep for 2, 4, 5, or 7 days. For each time point RNA was isolated from a pool of three to five ticks. Three replicates were obtained for each data point. Ovaries from *R*. *parkeri*-infected and non-infected adult female ticks were dissected from ticks that fed on sheep for 2, 4, 5, or 7 days. A pool of three to five ticks was used for each individual time point.

Ticks were dissected within 4 h after removal from the sheep in ice-cold 100 mM 3-(N-Morpholino)-propanesulfonic acid (MOPS) buffer containing 20 mM ethylene glycol bis-(β-aminoethyl ether)-N, N, N′, N′-tetraacetic acid (EGTA), pH 6.8. After removal, each tissue was washed gently in the same ice-cold buffer. All other manipulations were carried out on the ice. The dissected tissues were preserved in RNAlater buffer and stored at −80°C for further RNA isolation.

### RNA isolation

RNA was isolated from salivary glands and ovaries of unfed and partially fed ticks using an Illustra RNAspin Mini (GE Healthcare, Little Chalfont, Buckinghamshire, United Kingdom). Briefly, the total RNA was eluted into nuclease-free water and the concentration of total RNA was determined using a Nanodrop spectrophotometer (Thermo Fisher Scientific, Wilmington, DE, United States) and stored at −80°C for further analysis (Karim et al., 2011, 2012). All samples were DNase I treated before conducting RNAseq.

### Illumina sequencing

RNA isolated from tick salivary glands and ovaries as described above was submitted to Otogenetics Corporation (Norcross, GA, United States) for RNA-seq assays. Otogenetics checked the integrity and purity of the RNA samples using Agilent Bioanalyzer and OD 260/280. Total RNA was treated with the Ambion GLOBINclear-Human Kit to deplete globin mRNA that may have resulted from the bloodmeal; the Clontech SmartPCR cDNA kit (Clontech Laboratories, Inc., Mountain View, CA, United States) was used to generate 1–2 μg of cDNA from 100 ng of total RNA. Restriction digestion was used to remove adaptor sequences and the resulting cDNA was fragmented using Covaris (Covaris, Inc., Woburn, MA, United States), profiled using Agilent Bioanalyzer, and subjected to Illumina library preparation using NEBNext reagents (New England Biolabs, Ipswich, MA, United States). Agilent Bioanalzyer 2,100 was used to assess the quality, quantity, and size distribution of the Illumina libraries. The libraries were then submitted for Illumina HiSeq2000 sequencing according to the standard operation. Paired-end reads were generated and checked for data quality using FASTQC (Fuente, 2017). The number of reads for each of the 38 libraries ranged from 42 to 72 million, and the average read length was 128 nucleotides (nt) (Supplementary Spreadsheet S1).

### Bioinformatic analysis

The resulting reads were trimmed of low quality reads and Illumina primer sequences using the program trim-galore^1^ that wraps the CutAdapt (Martin, 2011) and FastQC (Brown et al., 2017) programs. The clean reads were assembled in single-ended mode with the programs Trinity (Haas et al., 2013) and Abyss (Simpson et al., 2009) (with k values ranging from 25 to 95 in intervals of 10). The assemblies were compacted using the program CD-hit-est (Li and Godzik, 2006), using a cut-off limit of 98% identity. To obtain the coding sequences (CDS), the largest open reading frame (ORF) of each contig was extracted. CDS shorter than 201 bases were excluded. The putative starting methionine of each CDS was selected based on the identification of a signal peptide indicative of secretion (Nielsen, 2017) and/or a blast (Madden, 2013) similarity match to known proteins from the Swiss-prot (Bairoch, 2000) database and from the Arachnida protein sequences available on GenBank. If the starting methionine was not found, a non-methionine amino acid (aa) was chosen as the first aa of the truncated CDS (Benson, 2000). To identify the bacterial-derived transcripts, we compared by blastp the transcript translations to five strains of *R*. *parkeri* available in GenBank, as well as 513 proteomes from the genus Francisella, 24 from *Coxiella*, one of Candidatus *Midichloria mitochondrii* and 11 from *Anaplasma phagocytophilum*. The reads from each library were mapped to the CDS using the RSEM program using the Bowtie2 aligner with the sensitive setting, which reports the best score from a read seed of length 22, assigning negative score values for gaps and mismatches and taking in consideration the qual value of the reads. Supplementary Table S3 reports the number of reads obtained for each library and the number of mapped reads for each library according to the provenance of the transcript (Arachnida, Bacterial or Unknown) (Li and Dewey, 2011). Statistical analysis of differentially expressed genes (DEG) was done with EdgeR (Robinson et al., 2010). Heatmaps were done with the Gplots R package. The data is presented as a hyperlinked excel spreadsheet as previously described (Ribeiro et al., 2004).

## Results and discussion

### *Rickettsia parkeri* in ticks

**Figure 1:**
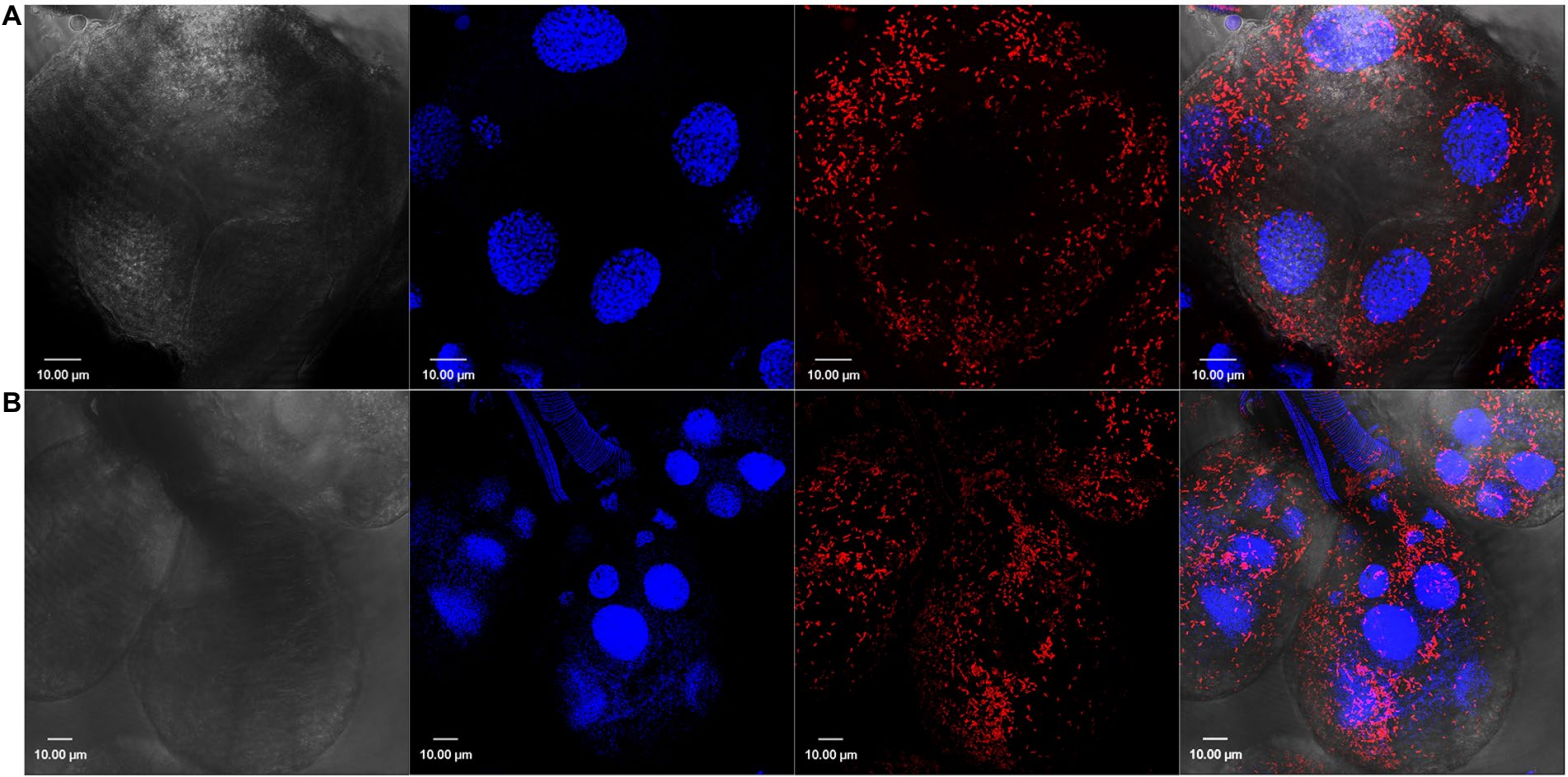
Confocal images showing tissue localization of *Rickettsia parkeri* in **(A)** unfed and **(B)** partially blood-fed *Amblyomma maculatum* salivary glands. Rod-shaped *rickettsiae* are localized intracellularly and depicted by their location in each salivary acini. DAPI (blue) was used to stain nuclei; *Rickettsiae*-specific antibody (M14-13) followed by Alexa-Fluor 568 conjugated anti-mouse IgG (red) was used to stain *R*. *parkeri* and differential interference contrast was used to capture tissue outline. Scale bar = 10 μM.

**Figure 2:**
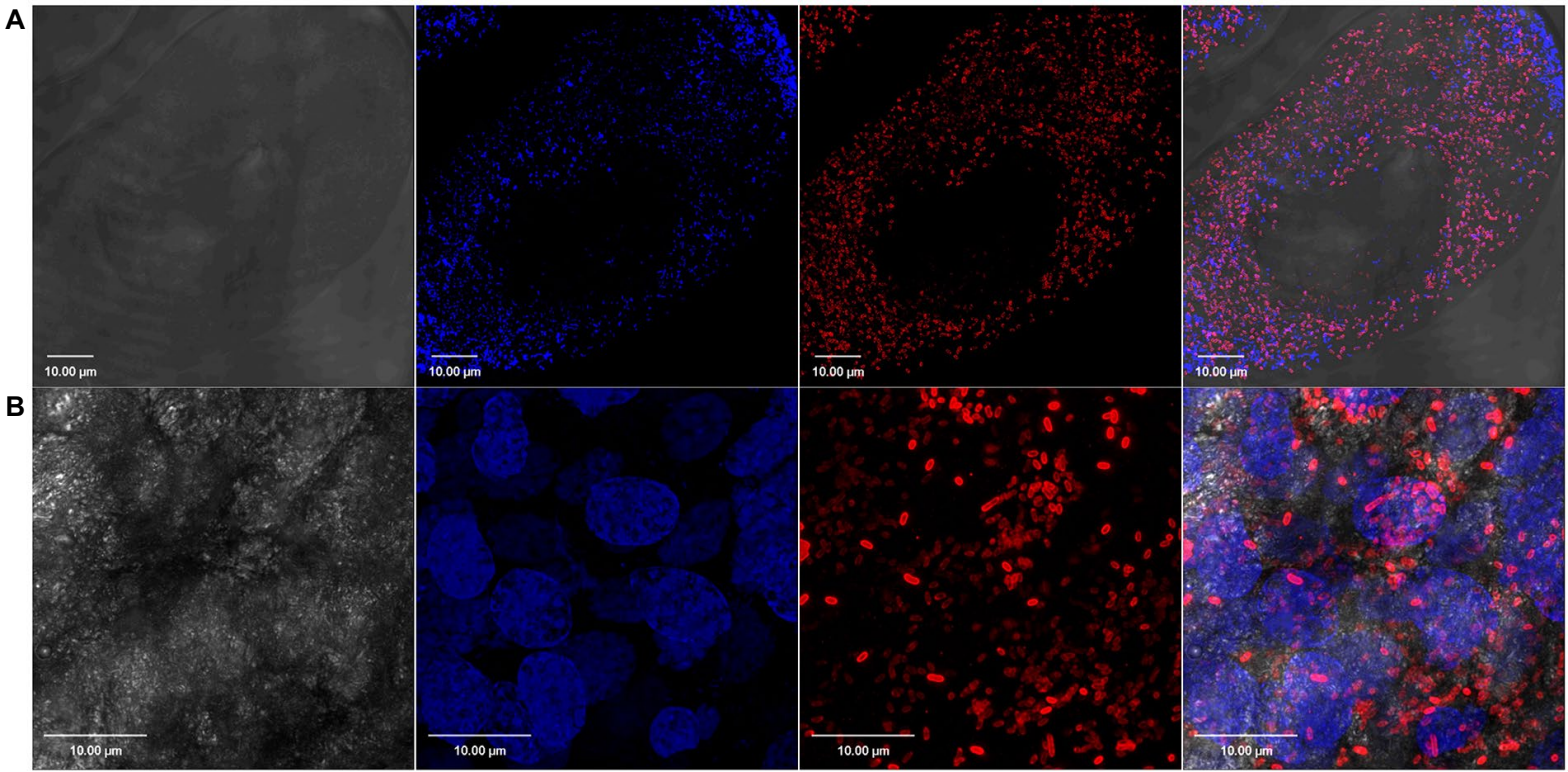
Confocal images showing tissue localization of *Rickettsia parkeri* in **(A)** unfed and **(B)** partially blood-fed *Amblyomma maculatum* ovaries. Rod-shaped *Rickettsiae* are localized intracellularly as depicted by their location in each oocyte. DAPI (blue) was used to stain nuclei; *Rickettsiae*-specific antibody (M14-13) followed by Alexa-Fluor 568 conjugated anti-mouse IgG (red) was used to stain *R*. *parkeri* and differential interference contrast was used to capture tissue outline. Scale bar = 10 μM.

We have previously demonstrated the presence of *R*. *parkeri* in *A*. *maculatum* salivary glands and ovaries by qPCR (Budachetri et al., 2018). In the current work, we confirmed this tissue distribution of *R*. *parkeri* using Rickettsia-specific antibodies. Confocal images showed intracellular localization of rod-shaped Rickettsiae in the unfed and partially fed salivary gland and ovarian tissues (Figures 1, 2). We next isolated RNA from infected and non-infected tissues to analyze the transcriptional changes in *A*. *maculatum* salivary glands and ovaries in response to the pathogen infection.

### Transcriptomic analysis

Assembly of the 38 libraries from the salivary glands and ovaries of *R*. *parkeri* infected and non-infected adult female *A*. *maculatum* ticks under different blood engorgement regimens resulted in 66,578 CDS (coding sequences). After mapping the reads from each library to these transcripts, 45,459 had a minimum read value of 50 (Supplementary Spreadsheet S1). The following analyses were performed with this set of transcripts and will follow the statistical results from EdgeR which contrasted the several variables under study. Eight times or more underexpressed or overexpressed differentially expressed transcripts (DET) with a false discovery rate of 0.05 or less were further analyzed.

### Differential gene expression in the *Amblyomma maculatum* microbiome in response to *Rickettsia parkeri* infection

**Figure 3:**
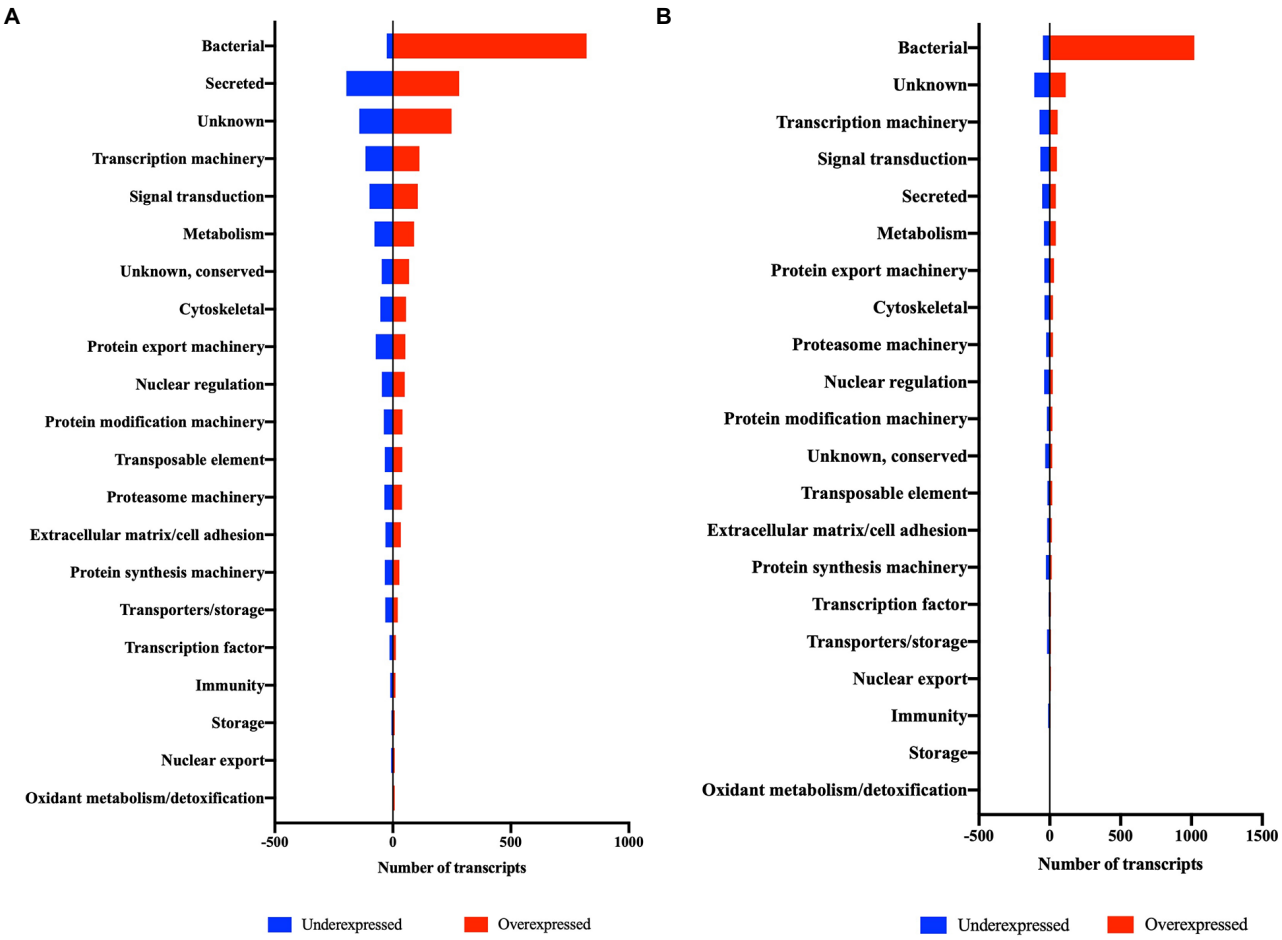
Differentially expressed transcripts (DET) of salivary glands **(A)** and ovary **(B)** of *R. parker*i-infected *A. maculatum* adult females in comparison to uninfected ticks grouped into functional categories.

Infection by *R*. *parkeri* altered the expression of genes of several functional categories in *A*. *maculatum* salivary glands and ovaries over the feeding course on a host (Table 1). Intriguingly, the bacterial category, comprising of transcripts from bacterial origin, was the most differentially expressed between *R*. *parkeri* infected and non-infected salivary glands and ovaries, corresponding to 25.9 and 47.9% of the total, respectively (Table 1 and Figure 3). Of the bacterial transcripts, the vast majority were overexpressed (97% in salivary glands and 95.5% in ovaries), indicating a high metabolic activity consistent with bacterial multiplication.

**Table 1:** Differentially expressed transcripts (DET– at least 8 fold over or underexpressed and with a false discovery rate of 0.05 or lower) from *A*. *maculatum* ovary and salivary glands in response to *R*. *parkeri* infection grouped into functional categories.

Functional category	Number of transcripts DET			
	Salivary glands		Ovary	
	Over	Under	Over	Under
Bacterial					821	26	1,020	48
Cytoskeletal	56	53	23	37
Oxidant metabolism/detoxification	8	2	2	4
Extracellular matrix/cell adhesion	34	31	15	18
Immunity	11	11	6	12
Metabolism	90	78	43	41
Nuclear export	8	7	8	5
Nuclear regulation	50	61	21	40
Protein export machinery	53	72	30	38
Protein modification machinery	41	38	19	21
Proteasome machinery	39	36	22	26
Protein synthesis machinery	28	34	13	27
Secreted	281	197	43	53
Signal transduction	106	99	50	66
Storage	8	6	2	4
Transposable element	40	34	17	17
Transcription factor	13	14	9	8
Transcription machinery	113	116	55	72
Transporters/storage	21	32	8	19
Unknown, conserved	69	47	17	32
Unknown	249	142	112	109
Total	2,139	1,136	1,535	697
Total over+under	3,275		2,232	

The microorganism primarily responsible for the differential expression of bacterial genes in *R*. *parkeri*-infected ticks was Candidatus Midichloria mitochondrii (CMM) (Table 2). Transcripts from CMM corresponded to 56.1 and 60.6% of the bacterial transcripts in salivary glands and ovaries, respectively. All CMM differentially expressed transcripts were overexpressed in *R*. *parkeri*-infected ticks, showing a positive gene modulation in response to the pathogen. CMM is a maternally-inherited endosymbiont identified in several hard tick species (Epis et al., 2008), including *A*. *maculatum* (Budachetri et al., 2018). CMM prevalence is variable, depending on the tick species and the life-stage analyzed (Cafiso et al., 2016; Duron et al., 2017). Its unique lifestyle allows the bacterium to reside not only in the tick cell cytoplasm but also in the mitochondrial inter-membrane space (Sassera et al., 2006). Our previous work has shown that 5-day *R*. *parkeri*-infected *A*. *maculatum* adult females had an increase in CMM levels in ovaries and salivary glands (Budachetri et al., 2018), suggesting a synergistic relationship between *R*. *parkeri* and the endosymbiont. The transcriptomic analysis described in the current study corroborated these results, showing a significant increase in the number of transcripts from CMM in salivary glands and ovaries of *R*. *parkeri*-infected ticks over the feeding course (Table 2).

**Table 2:** Bacterial differentially expressed transcripts (DET) in ovary and salivary glands in response to *R*. *parkeri* infection.

Bacterial genera	Number of transcripts DET			
	Salivary glands		Ovary	
	Over	Under	Over	Under
CMM					475	0	647	0
*Rickettsia parkeri*	207	3	183	3
Other *Rickettsia*	135	17	151	18
*Francisella sp.*	4	6	39	27
Total	821	26	1,020	48
Total over+under	847		1,068	

CMM, Candidatus Midichloria mitochondrii; Over, overexpressed; Under, underexpressed.

In adult females of the species *Ixodes ricinus*, CMM has been found in all individuals examined (Sassera et al., 2006), being identified in high numbers as the dominant bacterium in the tick ovary (Guizzo et al., 2020). In contrast, CMM was present in low levels in *A*. *maculatum* ovary but multiplied in response to infection by R. *parkeri* (Budachetri et al., 2018). Low levels of CMM have been identified in I. ricinus salivary glands (Olivieri et al., 2019). In *A*. *maculatum* CMM was also found in low numbers, but expanded significantly in response to *R. parkeri* infection (Budachetri et al., 2018). CMM is suggested to act as a nutritional symbiont due to its ability to synthesize the B vitamins biotin (B7) and folic acid (B9) (Sassera et al., 2011). Obligate hematophagous arthropods are presumed to rely on symbionts to obtain micronutrients deficient in host blood that are essential to their development and fitness (Rio et al., 2016; Duron and Gottlieb, 2020). Our transcriptomic analysis showed that a CMM putative biotin synthase (transcript ID 7830) and a putative folate metabolism protein (transcript ID 1034) were overexpressed in *R*. *parkeri*-infected *A*. *maculatum* ticks in both salivary glands and ovaries. This indicates that CMM from *A*. *maculatum* as well as CMM identified in I. ricinus may encode genes for the biosynthetic pathways of B vitamins. Even though CMM has been identified as part of the indigenous microbiome of *A*. *maculatum* (Budachetri et al., 2018), the transcripts implicated in B vitamin synthesis were only expressed in *R*. *parkeri*-infected ticks. This suggests that CMM does not play a role as an essential B vitamin provider in this tick life-stage. Interestingly, a study showed that *A*. *maculatum R*. *parkeri*-free larva and nymph molting success was affected negatively in comparison to *R*. *parkeri*-infected ticks, indicating a positive effect of the pathogen on tick fitness (Wright et al., 2015). These observations led us to hypothesize that the increase in CMM numbers in infected ticks could confer a metabolic advantage, providing an extra support of vitamins to the tick, improving its fitness, and benefiting the pathogen indirectly. Biotin and folic acid are cofactors of several biological reactions that could be involved in pathogen persistence within the tick, transovarial and transstadial transmission between ticks, or the transmission to the vertebrate host. The physiological role of CMM in other *A*. *maculatum* life-stages and its interaction with *R*. *parkeri* and other pathogens remains to be elucidated and could reveal important insights into the non-pathogen-pathogen-tick interaction.

**Figure 4:**
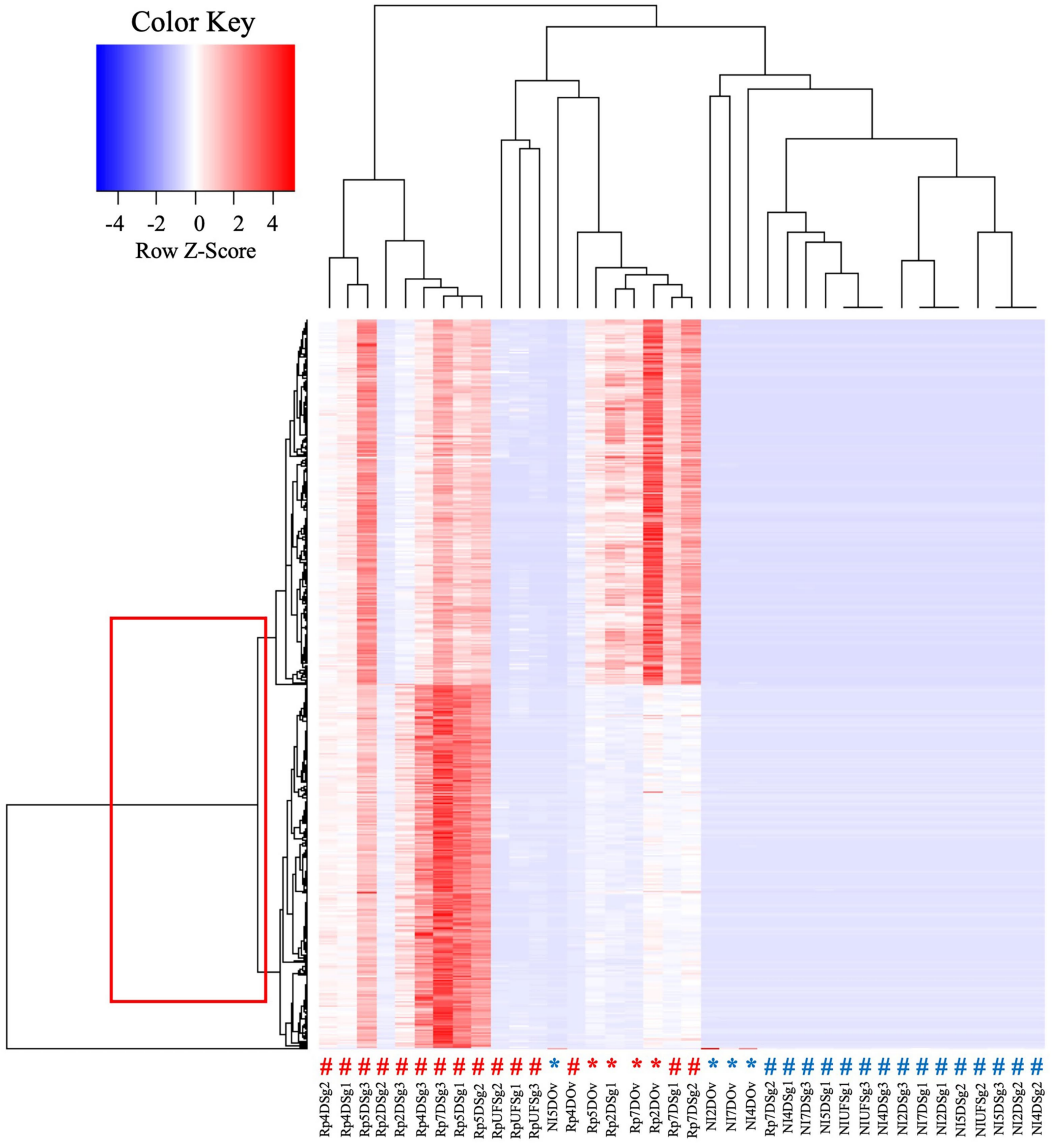
Heat map analysis of transcripts from CMM in salivary glands and ovaries of *R. parkeri*-infected and non-infected *A. maculatum* ticks. The box indicates two clusters of transcripts. The # symbols indicate results for salivary gland transcripts, and the * indicate those for ovary transcripts, their red color indicating results for *R*. *parkeri* infected and the blue symbols for non-infected ticks. Rp, *Rickettsia parkeri*-infected; NI, non-infected; Sg, salivar glands; Ov:ovary; D, day of feeding. A more detailed description of the experimental condition of each sample can be found in the Supplementary Spreadsheet S3.

Intriguingly, the heat map analysis of transcripts from CMM in ovaries and salivary glands revealed two clusters, suggesting that there were two lineages of CMM in *A*. *maculatum* (Figure 4). Within the salivary glands of blood-fed *R*. *parkeri*-infected ticks the two strains of CMM were identified in most of the samples over the feeding course, while in the ovaries Cluster 2 was absent. Cluster 1, the most abundant lineage in the salivary glands, was 90% similar to CMM sequences deposited in GenBank while Cluster 2 was only 73% similar. Transcripts from both CMM clusters were overexpressed in *R*. *parkeri* infected ticks, suggesting a similar pattern of regulation of microbial growth regardless of the CMM strain. Three different lineages of CMM have already been described in association with ticks (Buysse and Duron, 2018). They present distinct evolutionary strategies: two of them are more generalists, being acquired horizontally between tick species, and the other one has a high degree of specificity to the Ixodes genus (Buysse and Duron, 2018). This variable degree of interdependency suggests that CMM might affect tick hosts differentially. Further studies are needed to elucidate the nature of the interactions between each CMM lineage and *A*. *maculatum* ticks. Of note, this study represents the first description of two potential lineages of CMM coexisting within the same tick species.

As expected, *R*. *parkeri* genes were identified as differentially overexpressed in infected ticks (Table 2). The pathogen infects the tick ovary and salivary glands contributing to both vertical and horizontal transmission (Budachetri et al., 2018). Transcripts from other Rickettsia species were also identified as largely overexpressed in ovary and salivary glands (Table 2). Bacteria from the genus Rickettsia have already been described either as tick pathogens or endosymbionts (Kurtti et al., 2016; Hensley et al., 2021). The non-pathogenic Candidatus Rickettsia andeanae has been identified in *A*. *maculatum* salivary glands and ovaries (Lee et al., 2019). The co-infection of Candidatus *R*. *andeanae* and *R*. *parkeri* resulted in increased levels of the pathogen, suggesting a synergic interaction (Lee et al., 2019). *Rickettsia buchneri* is the main symbiont of *Ixodes scapularis*, being vertically transmitted and capable of encoding genes for the biosynthetic pathway of the B vitamin biotin (Gillespie et al., 2012; Kurtti et al., 2015). In *R*. *parkeri*-infected ticks, transcripts from Rickettsias other than *R*. *parkeri* followed the same expression profile as CMM, being markedly overexpressed in response to the pathogen. A transcript for folate metabolism protein (Transcript ID 9558) expressed by a *Rickettsia* sp. was found overexpressed in ovary and salivary glands of *R*. *parkeri*-infected ticks, suggesting that it could act as a nutritional endosymbiont. However, was observed for CMM, the folate gene was only expressed in *R*. *parkeri*-infected ticks. This suggests that the bacterium does not play a nutritional role in this tick life-stage, while strengthening the hypothesis that there is a synergic interaction between non-pathogenic-pathogenic Rickettsiales.

Francisella constitutes an important fraction of the *A*. *maculatum* microbiome (Budachetri et al., 2014). Francisella species have been described as endosymbionts in several tick species (Binetruy et al., 2020; Buysse et al., 2021, 2022). In Ornithoros moubata, a Francisella bacterial species was shown to play a role as a nutritional symbiont, being essential for the tick development (Duron et al., 2018). Nevertheless, the role of other Francisellas in their tick hosts remain unexplored. Among the differentially expressed transcripts from bacteria, those from Francisella appeared to be the least altered in response to *R*. *parkeri* infection. This corroborated previous results showing that Francisella did not multiply in 5-day adult females salivary glands and ovaries in *R*. *parkeri* infected-ticks (Budachetri et al., 2018).

Together, these results showed that the *A*. *maculatum* non-pathogenic microbiome was differentially modulated in the presence of *R*. *parkeri*. While transcripts from CMM and of the genus Rickettsia were largely overexpressed in response to the pathogen, transcripts from Francisella were only slightly affected. It is interesting to point out that the infection by *R*. *parkeri* selectively led to an increase in the numbers of non-pathogenic Rickettsiales, such as CMM and other bacteria from the genus Rickettsia. We hypothesized that the synergic interaction between Rickettsiales could favor the pathogen’s transovarial and transstadial transmission through an improvement in tick fitness due to the B vitamins provided by CMM and Rickettsia sp. Alternatively, the expansion in non-pathogenic Rickettsiales numbers could benefit *R*. *parkeri* perpetuation by playing a role in masking the pathogen from tick immune recognition. Tick endosymbionts are assumed to be tolerated by the tick immune system due to the biological advantage they confer on host fitness. Therefore, the extra support of vitamins provided by the non-pathogenic Rickettsiales could confound the tick immune system allowing the multiplication of all Rickettsiales, including the pathogenic *R*. *parkeri*. More studies are needed to test this hypothesis and fully elucidate the interaction between the *A*. *maculatum* non-pathogenic microbiome and *R*. *parkeri*.

### Differentially expressed tick genes in the salivary glands of *Rickettsia parkeri*-infected *Amblyomma maculatum*

Even though bacterial genes represented a large fraction of the differentially expressed genes in response to the *R*. *parkeri* infection, several functional categories of tick genes were also affected by the presence of the pathogen (Table 1; Figure 3). A similar number of transcripts were underexpressed and overexpressed in most functional categories, showing a global dysregulation of the tick metabolism in response to the pathogen. Intriguingly, while transcriptomic analysis of tick tissues in response to pathogen infection frequently display a marked overexpression of transcripts involved in tick host defense (Mulenga et al., 2003; Rudenko et al., 2005; Heekin et al., 2013; Kim et al., 2018; Martins et al., 2019), the *A*. *maculatum* infection by *R*. *parkeri* caused a phenomenon that we described as a “balanced dysregulation” of the tick metabolism.

*Rickettsia parkeri* infection resulted in the overexpression of a slight majority (54.3%) of diferentially-expressed tick transcripts detected in the salivary glands. The functional category comprising secreted transcripts was the main component responsible for the observed overexpression, representing 21.3% of the overexpressed transcripts. Tick infection by pathogens has previously been correlated with alterations in the expression of salivary secreted transcripts (Ribeiro et al., 2006; Mercado-Curiel et al., 2011; Martins et al., 2019; Paulino et al., 2021). *Metalloproteases*, *evasins*, *Lipocalins*, *mucins*, and protease inhibitors were some of the secreted proteins found in this study to be differentially modulated in response to *R*. *parkeri* infection (Table 3). Salivary transcripts have also been shown to be differentially expressed in *A*. *americanum* ticks infected by the Rickettsial pathogen Ehrlichia chaffeensis (Kim et al., 2018). Interestingly, while in *R*. *parkeri*-infected ticks most of the transcripts for lipocalins were overexpressed (Table 3), in *E*. *chaffeensis*-infected ticks they were underexpressed (Kim et al., 2018). Proteins from this family were also underexpressed in *Amblyomma aureolatum* ticks infected by *R*. *rickettsii* (Martins et al., 2019). Lipocalins counteract vertebrate host defenses by playing an anti-inflammatory role through the binding of small hydrophobic molecules, such as histamine, at the tick bite site (Valdés, 2014). In addition to lipocalins, metalloproteases were found to be underexpressed in *E*. *chaffeensis*-infected *A*. *americanum* ticks (Kim et al., 2018), but were overexpressed in *R*. *parkeri*-infected ticks (Table 3). Vaccination and gene silencing experiments have shown that salivary metalloproteases contribute to the blood feeding success in several tick species (Decrem et al., 2008; Imamura et al., 2009; Ali et al., 2015).

**Table 3:** Secreted differentially expressed transcripts (DET– at least 8 fold over or underexpressed and with a false discovery rate of 0.05 or lower) from *A*. *maculatum* salivary glands in response to *R*. *parkeri* infection grouped into functional categories.

Functional category	Number of transcripts DET Salivary glands	
	Over	Under
Metalloproteases			39	17
Evasins	26	18
Lipocalins	55	50
Mucins	27	22
Protease inhibitors	17	16

Over, overexpressed; Under, underexpressed.

Salivary mucins and evasins were also overexpressed in response to *R*. *parkeri* infection (Table 3). Proteins from these families were identified as components of the salivary gland and saliva of different tick species (Hayward et al., 2017; Ribeiro et al., 2017; Tirloni et al., 2020). Mucins are assumed to help to lubricate tick mouthparts facilitating the blood acquisition (Francischetti et al., 2008), while evasins bind host chemokines, inhibiting the inflammatory response of the host through the recruitment of leukocytes (Hayward et al., 2017). While several evasins were found to be differentially expressed in this study, with the majority being overexpressed, only one underexpressed transcript for evasin was found in the sialotranscriptome of *Rhipicephalus microplus* infected by the intracellular protozoan Theileria equi (Paulino et al., 2021). The overexpression of the salivary transcripts for lipocalins, metalloproteases, mucins and evasins induced by *R*. *parkeri* infection suggest that the pathogen can modulate the expression of tick salivary factors to facilitate its propagation by guaranteeing a successful host blood feeding. The second most overexpressed category was that of transcripts with unknown function, representing 18.9% of the total. However due to the lack of similarity with the analyzed databases, we cannot predict their function. Our results demonstrate that pathogen infection induced the expression of novel tick salivary transcripts, which may play a role in *R*. *parkeri* persistence and transmission.

Although pathogens have the ability to evade the immune system to survive within the tick host, immune transcripts are frequently overexpressed in tick salivary glands in response to pathogen infection (Kim et al., 2018; Martins et al., 2019; Paulino et al., 2021). It is assumed that the tick immune system is activated to limit pathogen numbers to levels that are not harmful to the host. Interestingly, in the salivary glands of *R*. *parkeri*-infected ticks, the same number of immune transcripts were underexpressed and overexpressed (Table 1), indicating a balanced dysregulated tick immune response to the infection. This suggests a modulation of the tick immunity, allowing the pathogen to survive without negatively impacting tick fitness. Other functional classes typically modulated positively or negatively in response to pathogen infection were balanced, in accordance with a dysregulated gene expression in *R*. *parkeri*-infected *A*. *maculatum* (Table 1). From the 33 differentially expressed transcripts grouped into the protease inhibitors functional class, a similar number was underexpressed and overexpressed (Table 3). Out of these, 24 were transcripts for Kunitz inhibitors, which also exhibited a balance of under-expressed and over-expressed (Table 3). An overall negative or positive modulation of Kunitz inhibitors, depending on the tick-pathogen interaction analyzed, has been shown in other reports (Kim et al., 2018; Martins et al., 2019). Protease inhibitors of Kunitz type are classically described as anti-coagulants, facilitating blood uptake (Corral-Rodríguez et al., 2009). Alternatively, a Kunitz inhibitor of Dermacentor variabilis was demonstrated to exert a bacteriostatic effect against Rickettsia montanensis (Ceraul et al., 2008). The balanced dysregulation of Kunitz inhibitors in response to *R*. *parkeri* infection could be part of a pathogen compensatory mechanism enabling *A*. *maculatum* successful blood feeding and/or pathogen survival without major alterations in tick metabolism. The remodeling of the tick cytoskeleton has been described as part of pathogens’ mechanisms to establish infection in both tick and vertebrate host (de la Fuente et al., 2016). In the salivary glands of adult female *I*. *scapularis* ticks infected by *Anaplasma phagocytophilum*, transcripts for components of the cytoskeleton were differentially expressed (Fuente, 2017). In our study a similar number of transcripts for tick cytoskeleton proteins were underexpressed and overexpressed (Table 1), showing a balanced dysregulated modulation of these tick genes in response to *R*. *parkeri* infection. This balanced dysregulation could facilitate tick cell invasion by the pathogen without considerably affecting the tick’s regular metabolism.

### Differentially expressed tick genes in the ovaries of *Rickettsia parkeri*-infected *Amblyomma maculatum*

In the ovaries, several *A*. *maculatum* transcripts were differentially expressed in response to *R*. *parkeri*. In contrast to our findings in salivary glands, the pathogen caused a slight underexpression of the total transcripts (55.7%), across several functional categories (Table 1). It has been shown that tick pathogens that are transovarially transmitted, such as *R*. *parkeri*, cause an infection-related differential expression of tick proteins (Mulenga et al., 2003; Rachinsky et al., 2007; Heekin et al., 2013; Antunes et al., 2019). In R. microplus ovarian genes involved in the immune system, detoxification, and stress response were found to be differentially modulated in response to infection by *Babesia bovis*. (Heekin et al., 2013). The *A*. *maculatum* transcriptional response to *R*. *parkeri* infection, however, was distinct from those previously described. While immune-related transcripts for microplusin and defensins were overexpressed in the ovaries of *B*. *bovis*-infected *R*. *microplus* (Heekin et al., 2013), transcripts categorized as immune-related were found in our study to be mostly underexpressed in the ovaries of *R*. *parkeri*-infected ticks (Table 1). Immune transcripts were also overexpressed in the ovary of *D*. *variabilis* ticks infected by *Ricketsia montanensis* (Mulenga et al., 2003). We speculate that the downregulation of the *A*. *maculatum* ovarian immune response could facilitate vertical transmission of the pathogen, guaranteeing their successful persistence in tick population and reducing their dependence of reservoir hosts.

Moreover, Kunitz inhibitors, which are assumed to act as components of the ovarian immune response against pathogens, were overexpressed in the ovaries of *R*. *microplus* infected by *B*. *bovis* (Rachinsky et al., 2007; Heekin et al., 2013). In our analysis, two transcripts for Kunitz inhibitors were found differentially expressed in the ovaries in response to *R*. *parkeri* infection (Supplementary Spreadsheet S2). One of them was underexpressed and the other one overexpressed, corroborating to the suggested balanced dysregulated nature of the tick-pathogen interaction. A similar transcriptional modulatory profile was found for heat-shock proteins. While ovarian heat-shock proteins were induced in *R*. *microplus* and *Rhipicephalus annulatus* in response to *B*. *bovis* and *B*. *bigemina*, respectively (Heekin et al., 2013; Antunes et al., 2019), in *R*. *parkeri*-infected *A*. *maculatum* the same number of transcripts for these proteins were underexpressed and overexpressed (Supplementary Spreadsheet S2). Heat-shock proteins are stress response-related proteins induced as the result of protein misfolding caused by infection (Hernandez et al., 2019). Along with other tick molecules, heat-shock proteins play a role in limiting pathogen numbers (Sonenshine and Macaluso, 2017). The transcriptional regulatory profile for Kunitz inhibitors and heat-shock proteins falls into the suggested balanced dysregulation rule and could reflect compensatory mechanisms of the tick metabolism to the well-established *R*. *parkeri* infection.

While several transcripts for cytochrome P450 were overexpressed in response to B. bovis infection in *R*. *microplus* (Heekin et al., 2013), in our study they were not differentially expressed. Other transcripts related to detoxification such as glutathione S-transferase, superoxidase dismutase and peroxinectin were induced by *B*. *bovis* infection (Heekin et al., 2013), but do not appear differentially expressed in our analysis. Similarly, a transcript for glutathione S-transferase was found overexpressed in the ovaries of *D*. *variabilis* infected by *Ricketsia montanensis* (Mulenga et al., 2003). Detoxification/oxidation molecules are suggested to be involved with the host protection against the oxidative stress caused by pathogen infection (Hernandez et al., 2019). In contrast to previous reports, our results show that infection by *R*. *parkeri* does not considerably affect the tick detoxification metabolism. Indeed, only six transcripts for the functional category of detoxification/oxidation were differentially expressed in *R*. *parkeri*-infected ticks, most of which were underexpressed (Table 1). We hypothesize that the underexpression of tick transcripts involved in detoxification and oxidation metabolism, in association with the underexpression of tick immune-related transcripts, may represent a mechanism by which the pathogen alters host transcription to favor perpetuation and vertical transmission. Nevertheless, it is important to point out that a small fraction of tick pathogens are vertically transmitted, and the number of studies focused on tick ovary transcriptional/proteomic modulation in response to vertically transmitted pathogens is still scarce. Further investigation of additional tick-pathogen associations could contribute to the understanding of the mechanisms by which specific pathogens manipulate tick metabolism to facilitate vertical transmission.

In both analyzed organs the presence of *R*. *parkeri* caused a balanced dysregulated expression of tick transcripts from most of the functional classes. While the majority of the reports describing transcriptomic analyses of ticks infected by pathogens have analyzed the tick metabolic responses to a novel infection (Kim et al., 2018), the ticks analyzed in this study were from an established *R*. *parkeri*-infected colony maintained in association with the pathogen over generations. As a consequence, the typical overexpression of transcripts involved with tick host defenses that occurs in response to the first encounter with a pathogen was not observed in this long-term tick-pathogen interaction. We speculate that the balanced dysregulation of the differentially expressed transcripts observed in this study could be the result of compensatory mechanisms of a well-adapted tick-pathogen interaction. This may explain how the pathogen is able to survive and continue transmission without significant deleterious effects to the development and fitness of the tick host.

## Conclusion

In this study, we performed a transcriptomic differential analysis of *A*. *maculatum* ovaries and salivary glands in response to *R*. *parkeri* infection. The results revealed that bacterial genes were the most differentially expressed. This is the result of an expansion in non-pathogenic and pathogenic Rickettssiales numbers over the feeding course on the host. Tick genes were also modulated in response to the bacterial infection, being slightly overexpressed in the salivary glands and underexpressed in the ovaries. A balanced dysregulation of tick metabolism in response to *R*. *parkeri*-infection is suggested, reflecting a well-adapted tick-pathogen interaction.

While in the salivary glands a slight overexpression of transcripts involved in blood acquisition could facilitate pathogen propagation, in the ovaries the underexpression of immune transcripts may contribute to the pathogen’s vertical transmission. In addition, we speculate that there is a relationship of cooperation between tick and pathogen with mutual beneficial effects to both species, due to the significant increase in endosymbiont levels. The extra support of vitamins provided by the non-pathogenic Rickettssiales has the potential to improve tick fitness, benefiting *R*. *parkeri* perpetuation indirectly. This suggests an orchestrated synergic tripartite interaction between tick, pathogen, and endosymbionts. The generation of an *A*. *maculatum* germ-free tick line could provide further insights into this complex relationship, revealing intriguing aspects of the role played by endosymbionts in pathogen-infected and non-infected ticks.

## Data availability statement

The datasets presented in this study can be found in online repositories. The names of the repository/repositories and accession number(s) can be found in the article/Supplementary material.

## Ethics statement

The animal study was reviewed and approved by the Institutional Animal Care and Use Committee at the University of Southern Mississippi approved all the protocols (#15101501.2 and 17191206.1) before the experiments commenced.

## Author contributions

JR and SK: conceptualization, formal analysis, project administration, resources, supervision, writing-review, and editing. KB, AA, JR, and SK: data curation, investigation, and methodology. SK: funding acquisition. AA and SK: validation. MG, JR, and SK: visualization. MG: writing original draft. All authors contributed to the article and approved the submitted version.

## Funding

This research was principally supported by USDA NIFA award # 2017–67017-26171, the Mississippi INBRE (an Institutional Award (IdeA) from the National Institute of General Medical Sciences of the National Institutes of Health under award P20GM103476). JR and MG were supported by the Intramural Research Program of the National Institute of Allergy and Infectious Diseases (Vector-Borne Diseases: Biology of Vector Host Relationship, Z01AI000810-21. This work utilized the computational resources of the NIH HPC Biowulf cluster (http://hpc.nih.gov). The funders played no role in the study design, data collection, and analysis, decision to publish, or manuscript preparation.

## Conflict of interest

The authors declare that the research was conducted in the absence of any commercial or financial relationships that could be construed as a potential conflict of interest.

## Publisher’s note

All claims expressed in this article are solely those of the authors and do not necessarily represent those of their affiliated organizations, or those of the publisher, the editors and the reviewers. Any product that may be evaluated in this article, or claim that may be made by its manufacturer, is not guaranteed or endorsed by the publisher.

## Footnotes

^1^ https://github.com/FelixKrueger/TrimGalore
